# Characterization of the complete mitochondrial genome of *Aphyocypris chinensis* (Cypriniformes, Xenocyprididae), and its phylogenetic position in Cypriniformes

**DOI:** 10.1080/23802359.2021.1959460

**Published:** 2021-07-28

**Authors:** Sang Ki Kim, Hee-Wook Cho

**Affiliations:** Department of Zoology, Nakdonggang National Institute of Biological Resources, Sangju, South Korea

**Keywords:** *Aphyocypris chinensis*, mitochondrial genome, Cypriniformes, Xenocyprididae, phylogeny

## Abstract

*Aphyocypris chinensis* Günther, 1868 is a small freshwater fish of the family Xenocyprididae (Cypriniformes). In this study, we determined its complete mitochondrial genome and phylogenetic position in Cypriniformes. The complete mitochondrial genome is 16,608 bp in size, containing 13 protein-coding genes, two RNAs, 22 tRNAs, and a control region. It has the typical vertebrate mitochondrial gene arrangement. Our mitogenomic phylogeny revealed that *A*. *chinensis* belongs to Xenocyprididae, rather than Danionidae. This mitogenome information could play an essential role in resolving the conflict over its current taxonomic status in Cypriniformes.

*Aphyocypris chinensis* Günther, 1868 is a small freshwater fish in the family Xenocyprididae (Cypriniformes). It is widely distributed in China, far eastern Russia, Japan, and the Korean Peninsula, mainly from the Amur to Pearl River drainages (Chae et al. 2019; Watanabe et al. 2020). This species inhabits small ponds, creeks, and rice paddies. It has declined dramatically throughout much of its range in Korea and Japan, at least partly because of farmland improvement, including changes in irrigation management, and was nearly extinct before 1980 in Japan (Watanabe et al. 2020). The IUCN assessment assigned *A*. *chinensis* Least Concern status. In addition, the taxonomic placement of the genus *Aphyocypris* in Cypriniformes has undergone a considerable change (Huang et al. 2017; Schönhuth et al. 2018). Therefore, the complete mitogenome of *A*. *chinensis* was characterized and analyzed phylogenetically. We expect that the mitogenomic data will provide essential information for conserving biological resources and as a systematic key to *A*. *chinensis*.

The specimen examined was collected on 9 July 2020 by the Byeongseong River (36°17'15.48"N, 128°5'26.96"E), Sangju, South Korea, and deposited in the Nakdonggang National Institute of Biological Resources (URL: https://www.nnibr.re.kr, contact person: Sang Ki Kim and email: ivoice8324@gmail.com) under the voucher number NNIBR-P15439. Total genomic DNA was extracted from muscle tissue using a DNeasy Blood & Tissue Kit (QIAGEN, Hilden, Germany). The mitochondrial genome was sequenced using shotgun sequencing on a HiSeq 2000 platform (Illumina, San Diego, CA, USA) using libraries with 200 bp inserts and 100 bp paired-end sequencing. The DNA sequences were edited and assembled using Geneious v.2021.1.1. (Kearse et al. 2012) and deposited in GenBank (MZ018624). The tRNA genes were identified through their secondary structures using tRNAscan-SE ver. 2.0 (Lowe and Chan 2016). The protein-coding genes (PCGs), rRNA genes, and control region were identified through homology with previously sequenced *A*. *chinensis* collected from Fukuoka, Japan (NC_008650, Saitoh et al. 2006). The complete *A*. *chinensis* mitogenome was 16,608 bp in length, including two rRNA genes, 13 PCGs, 22 tRNA genes, a light-strand replication origin, and a putative control region. The overall base composition was 30.8% A, 28.1% T, 24.8% C, and 16.4% G, with a slight A + T content bias (58.9%). The relative positions and orientations of the genes were identical to those of most vertebrates. All of the mitochondrial genes except ND6 and eight tRNA genes are encoded on the heavy strand. The PCGs used two start codons (ATG and GTG) and four stop codons (TAA, TAG, TA, and T—). The 21 tRNA genes could fold into typical cloverleaf secondary structures, although tRNA^Ser^ (AGY) lacked the D-arm. The 12S and 16S rRNA genes located between rRNA^Phe^ and tRNA^Leu^ (UUR) were 957 and 1691 bp in length, respectively.

The phylogeny of cypriniform fishes was reconstructed using 27 mitogenomes. Bayesian inference phylogenetic inference was performed using MrBayes 3.2.7 (Ronquist et al. 2012) and IQ-TREE webserver (Trifinopoulos et al. 2016) for ML analysis. All 13 PCGs and two rRNA genes were aligned individually. The best-fit models of nucleotide substitution and partition schemes were selected using PartitionFinder2 (Lanfear et al. 2017). We followed recent classification studies (Stout et al. 2016; Huang et al. 2017; Schönhuth et al. 2018) and considered all families within the suborder Cyprinoidei. Our mitogenomic phylogeny (Figure 1) revealed that *A*. *chinensis* belongs to Xenocyprididae rather than Danionidae, *Aphyocypris* formed a distinct clade in Xenocyprididae, and *A*. *kikuchii* (NC_019620) was genetically close to *A*. *chinensis* (Korea and Japan), with insufficient genetic differentiation to be classified as distinct species.

**Figure 1.**
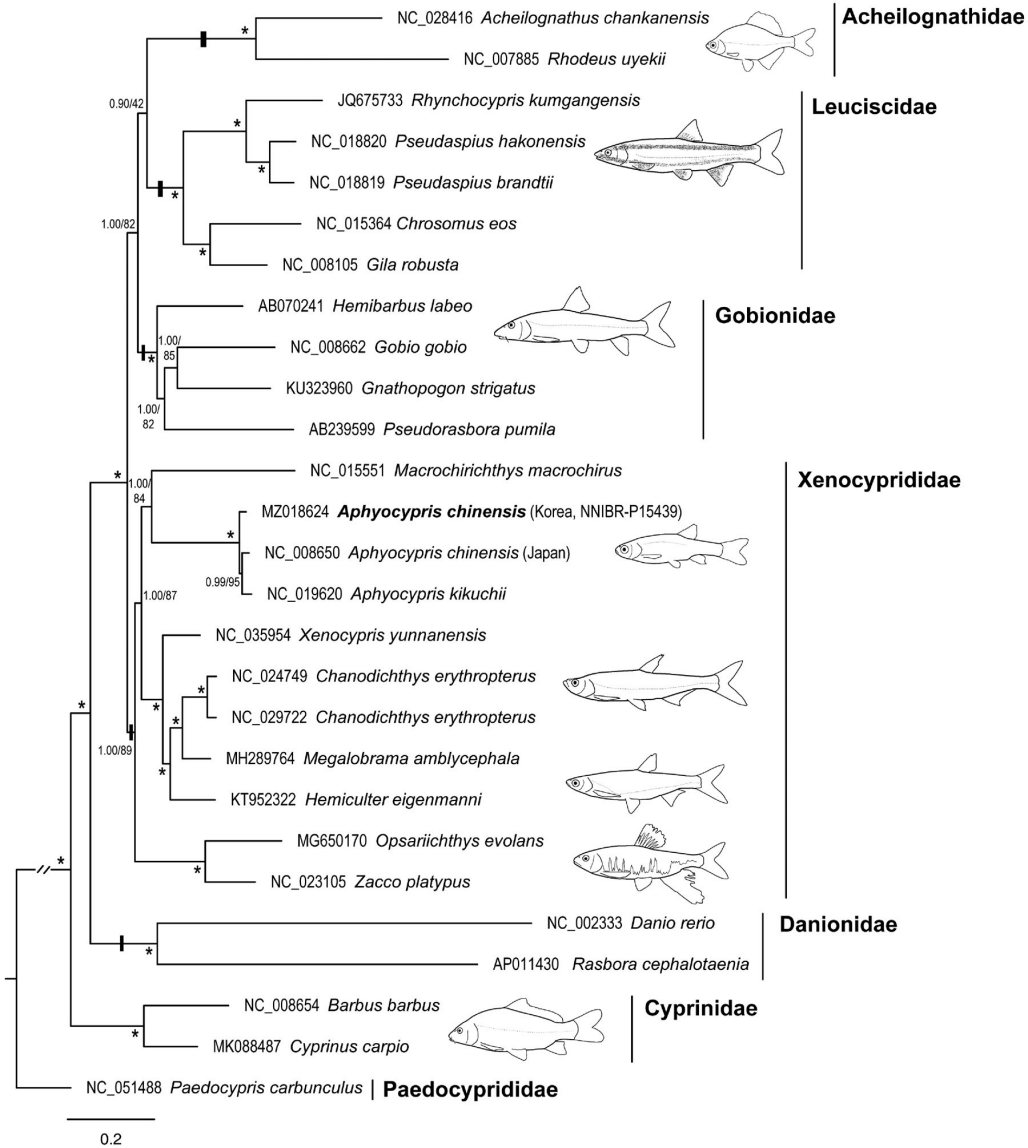
Bayesian inference tree of Cyprinoidei inferred from a mitochondrial dataset of 13 PCGs plus two rRNA genes. The values above and below the branches are the Bayesian posterior probability and maximum likelihood ultrafast bootstrap values, respectively. Values over 95% are represented by asterisks.

## Data Availability

The genome sequence data that support the findings of this study are openly available in GenBank of NCBI at https://www.ncbi.nlm.nih.gov under the accession no. MZ018624. The associated BioProject, SRA, and Bio-Sample numbers are PRJNA743720, SRR15042172, and SAMN20058879 respectively.

## References

[CIT0001] Chae BS, Song HB, Park JY. 2019. A field guide to the freshwater fishes of Korea. Seoul: LG Evergreen Foundation; p. 355.

[CIT0002] Huang S-P, Wang F-Y, Wang T-Y. 2017. Molecular phylogeny of the *Opsariichthys* group (Teleostei: Cypriniformes) based on complete mitochondrial genomes. Zool Stud. 56:e40. 31966239 10.6620/ZS.2017.56-40 PMC6517700

[CIT0003] Kearse M, Moir R, Wilson A, Stones-Havas S, Cheung M, Sturrock S, Buxton S, Cooper A, Markowitz S, Duran C, et al. 2012. Geneious Basic: an integrated and extendable desktop software platform for the organization and analysis of sequence data. Bioinformatics. 28(12):1647–1649. 22543367 10.1093/bioinformatics/bts199 PMC3371832

[CIT0004] Lanfear R, Frandsen PB, Wright AM, Senfeld T, Calcott B. 2017. PartitionFinder 2: new methods for selecting partitioned models of evolution for molecular and morphological phylogenetic analyses. Mol Biol Evol. 34(3):772–773. 28013191 10.1093/molbev/msw260

[CIT0005] Lowe TM, Chan PP. 2016. tRNAscan-SE On-line: integrating search and context for analysis of transfer RNA genes. Nucleic Acids Res. 44(W1):W54–W57. 27174935 10.1093/nar/gkw413 PMC4987944

[CIT0006] Ronquist F, Teslenko M, van der Mark P, Ayres DL, Darling A, Höhna S, Larget B, Liu L, Suchard MA, Huelsenbeck JP. 2012. MrBayes 3.2: efficient bayesian phylogenetic inference and model choice across a large model space. Syst Biol. 61(3):539–542. 22357727 10.1093/sysbio/sys029 PMC3329765

[CIT0007] Saitoh K, Sado T, Mayden RL, Hanzawa N, Nakamura K, Nishida M, Miya M. 2006. Mitogenomic evolution and interrelationships of the Cypriniformes (Actinopterygii: Ostariophysi): The first evidence toward resolution of higher-level relationships of the world's largest freshwater fish clade based on 59 whole mitogenome sequences. J Mol Evol. 63(6):826–841. 17086453 10.1007/s00239-005-0293-y

[CIT0008] Schönhuth S, Vukić J, Šanda R, Yang L, Mayden RL. 2018. Phylogenetic relationships and classification of the holarctic family Leuciscidae (Cypriniformes: Cyprinoidei). Mol Phylogenet Evol. 127:781–799. 29913311 10.1016/j.ympev.2018.06.026

[CIT0009] Stout CC, Tan M, Lemmon AR, Lemmon EM, Armbruster JW. 2016. Resolving Cypriniformes relationships using an anchored enrichment approach. BMC Evol Biol. 16(1):244. 27829363 10.1186/s12862-016-0819-5 PMC5103605

[CIT0010] Trifinopoulos J, Nguyen L-T, von Haeseler A, Minh BQ. 2016. W-IQ-TREE: a fast online phylogenetic tool for maximum likelihood analysis. Nucleic Acids Res. 44(W1):W232–W235. 27084950 10.1093/nar/gkw256 PMC4987875

[CIT0011] Watanabe K, Tabata R, Nakajima J, Kobayakawa M, Matsuda M, Takaku K, Hosoya K, Ohara K, Takagi M, Jang-Liaw N-H. 2020. Large-scale hybridization of Japanese populations of Hinamoroko, *Aphyocypris chinensis*, with *A. kikuchii* introduced from Taiwan. Ichthyol Res. 67(3):361–374.

